# 40 Hz vibrotactile stimulation ameliorates amyloid pathology and cognitive deficits in 5xFAD mice and is associated with alterations in Piezo1, ERK, and GSK-3β/p65 signaling

**DOI:** 10.1016/j.neurot.2026.e01077

**Published:** 2026-09-17

**Authors:** Myeong-Hyun Nam, Hee-Jung Park, Hee-Yeon Lee, Zu-Yu Chen, Hee-Deok Yun, Yuna Kim, Ju-Yeong Lee, Chang-Ho Shin, Jae-Young Ha, Young-Kwon Seo

**Affiliations:** a Department of Biomedical Engineering, Dongguk University, Goyang-si, 10326, Republic of Korea; b Department of AI Convergence Biomedical Engineering, Dongguk University, Goyang-si, 10326, Republic of Korea; c AriBio Co., Ltd., 56 Dongpangyo-ro, Bundang-gu, Seongnam-si, 13535, Republic of Korea

**Keywords:** Alzheimer's, Vibrotactile stimulation, Amyloid beta, Piezo1, GSK-3β, ERK pathway

## Abstract

40 Hz vibrotactile stimulation (VTS) is an emerging non-invasive therapy for Alzheimer's disease (AD), yet its specific mechanisms regarding amyloid-beta (Aβ) metabolism remain unclear. Six-month-old 5xFAD mice received daily 40 Hz VTS for four weeks. We assessed cognitive function, Aβ pathology, and underlying molecular pathways. VTS improved spatial learning and recognition memory, whereas no significant improvement was observed in short-term spatial working memory. VTS reduced hippocampal Aβ plaque burden and cortical soluble Aβ40 and Aβ42 levels, accompanied by decreased expression of APP, BACE1, and PS1 and increased expression of ADAM10 and IDE. It further attenuated neuroinflammation, oxidative stress and produced changes in cholinergic and synaptic plasticity-associated proteins. VTS increased hippocampal Piezo1 expression and ERK phosphorylation, increased inhibitory phosphorylation of GSK-3β at Ser9, decreased p65 phosphorylation, and reduced tau phosphorylation. 40 Hz VTS ameliorates several cognitive and neuropathological features of AD in 5xFAD mice. These improvements are associated with the modulation of Piezo1, ERK, and GSK-3β/p65 signaling pathways, highlighting its potential as a promising non-invasive AD therapeutic strategy.

## Introduction

Alzheimer's disease (AD), the most common neurodegenerative disorder in the elderly population, is characterized by impairments in memory and cognitive function [1,2]. The pathological hallmarks of AD include the formation of Aβ plaques and neurofibrillary tangles [3,4]. Amyloid beta (Aβ) is considered a key factor in the pathogenesis of AD, inducing neuroinflammation, neuronal damage, and synaptic loss [5,6].

Existing FDA-approved treatments for AD, such as donepezil and memantine, primarily provide symptomatic relief by inhibiting acetylcholinesterase (AChE) activity [7]. A hallmark of AD pathogenesis is the profound disruption of the cholinergic system, which has been identified as a primary contributor to cognitive decline [8]. This system's function is critically dependent on the balance between acetylcholine (ACh) synthesis, mediated by choline acetyltransferase (ChAT), and its degradation by acetylcholinesterase (AChE). More recently, therapeutic development has shifted focus toward fundamental treatments targeting the primary pathology. However, even recently approved drugs, such as lecanemab and donanemab, are associated with significant adverse effects, including cerebral edema and microhemorrhages [[9], [10], [11]]. Consequently, non-invasive methods are emerging as a promising therapeutic strategy to either complement or replace pharmaceuticals associated with such adverse effects.

Oxidative stress is a critical factor in AD pathogenesis, known to accelerate Aβ accumulation, tau hyperphosphorylation, and subsequent synaptic and neuronal loss [12]. Malondialdehyde (MDA), a key biomarker of lipid peroxidation, is consistently observed at elevated levels in 5xFAD mouse models [[13], [14], [15]]. Therapeutic interventions targeting oxidative stress have shown promise in various models. For instance, six weeks of whole-body vibration reduced MDA levels in the blood of women with fibromyalgia [16]. High-frequency repetitive transcranial magnetic stimulation (rTMS) effectively decreased MDA levels in a 3xTg AD mouse model, and TMS also reduced MDA in rats exposed to 3-nitropropionic acid-induced oxidative stress [17,18].

Gamma oscillations enhance synaptic plasticity and are closely associated with learning and memory processes. Studies have reported that gamma oscillations are reduced in association with the cognitive decline observed in AD, a phenomenon that has also been confirmed in various AD mouse models [[19], [20], [21], [22], [23]]. Based on these findings, recent research has focused on sensory stimulation as a strategy aimed at improving gamma oscillations [[24], [25], [26]]. Various neuromodulation techniques such as electrical, magnetic, sensory, and ultrasound stimulation have been investigated to determine their therapeutic effects on AD by inducing gamma oscillations. The results from these studies have shown positive outcomes, including improved memory, reduced Aβ load, and altered microglial activation responses. Furthermore, they mitigate neuronal and synaptic loss and provide neuroprotective effects [27,28].

While non-invasive sensory interventions, primarily visual and auditory 40 Hz stimulations, have shown promise, their clinical efficacy can be restricted by age-related visual or hearing impairments commonly observed in AD patients, as well as potential discomfort from flickering lights or continuous noise [29].

Recently, research utilizing vibrotactile stimulation (VTS) has been actively conducted. In studies where both Tau P301S and CK-p25 mice underwent 40 Hz, 1.01 m/s^2^ whole-body vibration (WBV) for 6 weeks, the results confirmed beneficial effects, including improved motor performance, synaptic preservation, and attenuation of neuronal loss [30]. Furthermore, another study utilizing aged rats (18–20 months old) demonstrated that 40 Hz WBV (Whole-Body Vibration) significantly improved cognitive function, modulated synaptic connectivity, and reduced neuronal apoptosis [31]. When we applied vibrotactile stimulation to a neurotoxicity-induced mouse model, we observed improved cognitive function, reduced oxidative stress, suppressed neuroinflammation, and enhanced synaptic plasticity.

Although VTS has shown significant therapeutic effects in animal models, its influence on Aβ accumulation and the precise underlying mechanisms are not fully understood. Recent studies have indicated that 40 Hz stimulation promotes a specific microglial phenotype, thereby enhancing clearance activity and mitigating neuroinflammation [28]. Another study reported that this frequency of stimulation reduces Aβ accumulation by decreasing amyloid precursor protein (APP) intermediates and suppressing inflammatory responses [24]. Nevertheless, the cellular and molecular processes that link VTS specifically to memory enhancement remain largely elusive.

In our previous study, we confirmed that vibrotactile stimulation activated the AKT/GSK-3β/β-catenin pathway, which led to improved cognitive function, reduced oxidative stress, suppressed neuroinflammation, and enhanced synaptic plasticity [32]. However, as this experiment was conducted using a neurotoxicity-induced model, we were unable to investigate its effects on Aβ accumulation. The 5xFAD mouse, one of the prominent AD animal models, develops significant Aβ accumulation and cognitive impairments, thereby exhibiting key pathological features similar to human AD. Using this mouse model, we investigated the effects of vibrotactile stimulation on Aβ accumulation, cognitive improvement, and neuropathological changes. The results from behavioral and molecular analyses of brain tissue revealed that vibrotactile stimulation improved cognitive function in the mice, suppressed Aβ production, and promoted its degradation. Furthermore, this reduction in Aβ attenuated neuroinflammation, mitigated cholinergic dysfunction, and improved synaptic plasticity.

## Materials and Methods

### Experimental animals

Male C57BL/6N mice were obtained from Dae Han Biolink Co., Ltd. (Eumseong, Republic of Korea). Six-month-old male 5xFAD mice (Stock No: 034848, The Jackson Laboratory, USA) were used as the AD model.

The 5xFAD mouse is a widely utilized animal model for AD research. These mice express five familial AD mutations: three in the *APP* transgene (Swedish (K670 N/M671L), Florida (I716V), and London (V717I)) and two in the *PS1* transgene (M146 L/L286V). Consistent with previous literature, cognitive impairments in this model are typically reported to manifest around 6 months of age [33].

All animals were maintained under controlled temperature (22 °C ± 2 °C) with a 12 h light–dark cycle. The mice were housed five per cage with free access to food and water and provided with environmental enrichment such as nesting materials. Animals were monitored daily for general health, body weight, and behavior.

The mice were divided into three groups: 1) **WT group** (Wild-type control, n = 8): C57BL/6Nmice. 2) **TG group** (Transgenic control, n = 8): 5xFAD mice. 3) **VIB group** (Vibration-treated, n = 8): 5xFAD mice treated with vibrotactile stimulation (acceleration 2.2 m/s^2^).

**Fig. 1:**
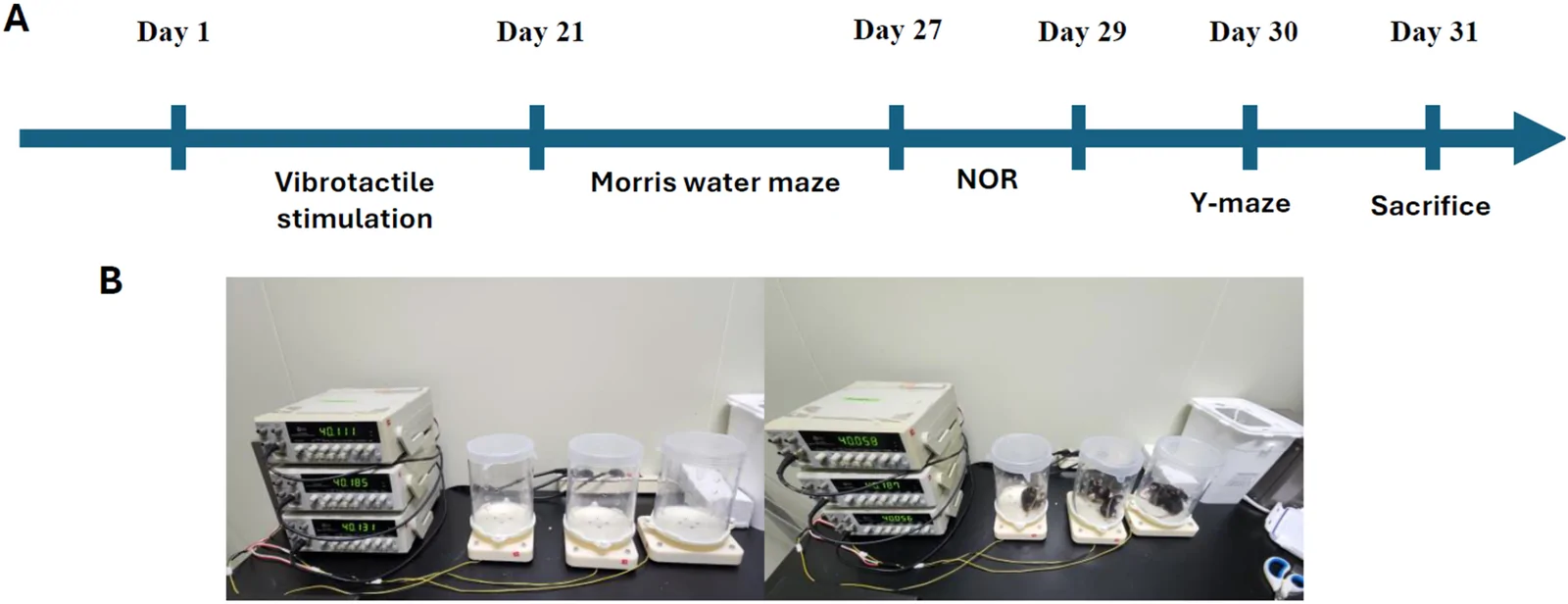
**Vibrotactile stimulation (VTS) apparatus and experimental schedule. (A**) Experimental timeline illustrating the VTS treatment and behavioral testing schedule. **(B)** Schematic of the VTS apparatus. A cylindrical plastic barrier was installed on the vibration plate to prevent mice from leaving. Each vibrator unit could stimulate up to three mice simultaneously.

The vibrotactile stimulation was administered for 4 weeks. All behavioral tests were conducted 30 min after the daily stimulation session. The overall experimental schedule has been organized (Fig. 1A).

To preclude potential confounding variables arising from estrous cycle-induced hormonal fluctuations on behavioral and molecular outcomes, this study exclusively utilized male mice. Consequently, we acknowledge that the generalizability of these findings to females remains a limitation of the current experimental design.

All animal experimental procedures were approved by the Institutional Animal Care and Use Committee of Dongguk University (IACUC- 2024-048-1).

### Vibrotactile stimulation procedure

A 40 Hz sine wave was supplied from a function generator (EZ Digital, FC-7002C, Anyang, Republic of Korea) to a circular vibrator with a maximum output of 5 W. A circular vibrating plate (12 cm in diameter) was designed to transmit the vibration to the entire body of the mice placed upon it. A cylindrical plastic barrier was installed around the plate to prevent the mice from moving off the vibrator (Fig. 1B). Up to three mice could be stimulated simultaneously on a single vibrator. For the VIB group, vibrotactile stimulation was applied daily at 1:00 p.m. for 30 min over a period of 4 weeks at an acceleration of 2.2 m/s^2^. To control the experimental environment, the mice in the WT and TG groups were placed on the same device for 30 min without receiving vibration stimulation. The acceleration value was measured prior to the stimulation sessions using an accelerometer (Dytran Instruments, Inc., 3225 F, Chatsworth, CA, USA) with a sensitivity of 10 mV/g, a measurement range up to 500 g, and a frequency response of 1.6–10,000 Hz (±10%).

## Behavior Tests

### Morris Water Maze (MWM) test

Spatial learning and memory were evaluated using the Morris Water Maze (MWM). The apparatus consisted of a circular, open-field pool (150 cm) filled with water, maintained at a constant temperature of 22 °C ± 1 °C. An automated video tracking system (ANY-maze) was employed to record and analyze all parameters, including swimming paths and latency times.

The pool was virtually divided into four equal quadrants. A hidden escape platform was submerged in the center of one target quadrant. For each trial, mice were introduced into the pool from the quadrant diagonal to the platform's location.

The protocol involved a 3-day acquisition phase, followed by a test session on day 4.During the acquisition phase, mice were given 60 s to locate the submerged platform. If a mouse failed to find the platform within this time limit, it was gently guided to it and allowed to remain there for a 30 s acquisition period. The probe test on day 4 followed an identical procedure, but this 30 s acquisition guidance was omitted.

Prior to the initiation of the main experimental procedures (i.e., vibration treatment and behavioral testing), all animals underwent prescreening to assess baseline swimming capability and general motor performance. This pre-assessment ensured that animals could be randomly assigned to experimental groups, balancing for baseline motor function. We rigorously standardized all experimental conditions to minimize potential stress-related variability. Throughout the MWM trials, all mice were monitored for swimming ability and motor coordination, and no inter-group differences were observed. Furthermore, the suitability of MWM for this paradigm is supported by previous studies that have reported valid cognitive outcomes following vibrotactile stimulation [31].

### Novel object recognition (NOR) test

Recognition memory was assessed using the Novel Object Recognition (NOR) test. The apparatus was a square, open-field chamber (25 cm × 25 cm × 25 cm) constructed from polyvinyl plastic.

The protocol consisted of three phases: 1) **Habituation:** Mice were individually placed in the empty chamber and allowed to explore freely for 5 min; 2) **Training (Familiarization) Trial:** Two identical objects were positioned within the chamber, and mice were allowed to investigate them for 5 min; 3) **Test Trial:** Following a 4-h inter-trial interval, one of the identical objects was replaced with a novel object. Mice were then reintroduced into the chamber for a 5-min test session.

The time spent actively exploring each object was recorded by the ANY-maze video tracking system. A Recognition Index (RI) was calculated to quantify the preference for the novel object using the following formula: Recognition Index = (Time spent with novel object)/(Total time spent exploring both objects) ∗100.

### Y-maze test

Short-term spatial working memory was evaluated using the Y-maze spontaneous alternation task. The apparatus was constructed of polyvinyl plastic and consisted of three identical arms (arm A, B, and C) oriented at 120° angles from each other.

Each mouse was initially placed in the central zone and permitted to explore the maze freely for 7 min. An arm entry was operationally defined as the point when the mouse's hind paws had completely entered the arm.

Spontaneous Alternation Score (SAS) was calculated as the percentage of successful alternations (i.e., consecutive entries into three different arms, e.g., ABC, BCA, CAB) relative to the total number of alternation opportunities (total arm entries − 2).

### Tissue preparation

Immediately after the final behavioral test, the mice were deeply anesthetized, blood was drawn, and their brains were removed for subsequent analyses, including biochemical analysis, Western blotting, and immunohistochemistry.

For the isolation of peripheral blood mononuclear cells (PBMCs), whole blood was collected into EDTA-treated tubes and gently shaken several times to prevent coagulation. Samples were centrifuged at 1000×*g* for 15 min at 4 °C. The upper plasma layer was aspirated and discarded, and the remaining cell pellet was resuspended in 5 mL of ACK lysis buffer and incubated for 5 min at room temperature to lyse red blood cells. To stop lysis, the suspension was transferred to a 50 mL conical tube and diluted with 45 mL of PBS. The solution was centrifuged again at 1000×*g* for 15 min at 4 °C. The supernatant was discarded, and the resulting PBMC pellet was collected for qRT-PCR analysis.

The whole cerebral cortex and hippocampus were harvested and immediately frozen in liquid nitrogen for biochemical analysis, qRT-PCR, and Western blotting. For histological analysis, mouse brains were fixed in 4% paraformaldehyde (PFA) overnight at 4 °C. The fixed tissues were then processed through a graded alcohol series for dehydration and subsequently embedded in paraffin.

### Lipid peroxidation (TBARS) assay

To quantify oxidative stress, lipid peroxidation levels were assessed by measuring malondialdehyde (MDA) concentrations using a Peroxidation (TBARS) Assay Kit (DoGenBio, Seoul, Republic of Korea). Briefly, isolated cerebral cortex tissue was homogenized in PBS containing heparin. The assay principle relies on the reaction of MDA, a primary product of lipid peroxidation, with thiobarbituric acid (TBA) to form a colorimetric MDA–TBA adduct.

The optical density (OD) of this adduct was measured at 540 nm. MDA concentrations were calculated by interpolation from a standard curve, which was prepared using a 2-fold serial dilution of an MDA standard (starting from 400 μM). All readings were performed in duplicate, averaged, and blank-subtracted. Final values were adjusted for any dilution factor and subsequently normalized to the total protein concentration, expressed as μM/mg protein.

### AChE activity assay

The enzymatic activity of acetylcholinesterase (AChE), the enzyme responsible for hydrolyzing acetylcholine, was quantified using the Amplite® Colorimetric Acetylcholinesterase Assay Kit (AAT Bioquest, Inc., Santa Clara, CA, USA). In accordance with the manufacturer's protocol, cortical tissue was homogenized in PBS.

For the assay, 50 μL of either sample or standard was combined with 50 μL of a working solution containing DTNB and acetylthiocholine. This mixture was incubated for 30 min at room temperature, allowing the enzymatic reaction to proceed. The absorbance was then measured at 410 nm. AChE activity was determined by interpolating from a standard curve, which was generated using 1:3 serial dilutions of a 1000 mU/mL AChE standard.

### Quantification of soluble amyloid-β 40 and amyloid-β 42 in the cerebral cortex by ELISA

Cortex tissues were homogenized in ice-cold PBS and centrifuged at 12,000×*g* for 15 min at 4 °C. The resulting supernatants, representing the PBS-soluble fraction, were collected, and Aβ40 and Aβ42 concentrations were quantified using commercially available ELISA kits (CUSABIO, Houston, TX, USA) according to the manufacturer's instructions. Aβ40 and Aβ42 levels were normalized to the total protein concentration of each sample.

### Quantitative analysis of the gene expression by qRT-PCR

Total RNA was isolated from both blood cells and hippocampal tissue using TRIzol reagent (Invitrogen, Waltham, MA, USA) according to the manufacturer's protocol. Briefly, phase separation was induced by adding 200 μL of chloroform (Sigma, St. Louis, MO, USA) to the tissue homogenate, followed by vigorous mixing and a 3-min incubation.

The samples were then centrifuged at 12,000×*g* for 15 min at 4 °C. The upper aqueous phase, containing the RNA, was carefully transferred to a new tube. RNA was precipitated by adding 500 μL of isopropanol, incubating for 10 min, and centrifuging at 12,000×*g* for 10 min.

After discarding the supernatant, the resulting RNA pellet was washed with 1 mL of 75% ethanol and collected by centrifugation at 7500×*g* for 5 min at 4 °C. The pellet was subsequently air-dried at room temperature and resuspended in 20 μL of ice-cold, RNase-free water. The concentration and purity of the extracted RNA were quantified using a NanoDrop spectrophotometer (Thermo Fisher Scientific, Waltham, MA, USA).

First-strand complementary DNA (cDNA) was synthesized from the total RNA using a reverse transcription master mix (Dynebio, Seongnam-si, Republic of Korea).

Quantitative real-time PCR (qRT-PCR) was performed to analyze gene expression levels using a StepOnePlus™ real-time PCR system (Applied Biosystems, Waltham, MA, USA) with TB Green® Premix Ex Taq™ (Takara Bio, Kusatsu, Japan). The thermal cycling protocol was initiated with an enzyme activation step at 95 °C for 20 s. This was followed by 45 cycles of amplification, consisting of denaturation at 95 °C for 3 s, annealing at 60 °C for 30 s, and extension at 72 °C for 30 s.

Upon completion of the amplification cycles, a melt curve analysis was conducted to verify product specificity. This involved denaturation at 95 °C for 15 s, annealing at 60 °C for 60 s, followed by a gradual temperature ramp (0.3 °C increments) to monitor temperature-dependent fluorescence dissociation.

This method was used to detect the expression of inflammation-related genes (TNF-α, IL-1β, IL-6) and apoptosis-related genes (Bax, Bcl-2). All primer sequences used for amplification are detailed in Table S1. Relative gene expression levels were quantified using the comparative CT (ΔΔCT) method.

### Western Blotting

For protein analysis, bilateral hippocampus were isolated and homogenized in sample buffer (0.25 M Tris-HCl pH 6.8, 4% SDS, 40% glycerol, 0.05% bromophenol blue, 10% β-mercaptoethanol). The resulting lysates were clarified by centrifugation at 12,000×*g* for 15 min at 4 °C, and the supernatants were collected. Total protein concentration was determined using a BCA assay against a standard curve generated with bovine serum albumin (BSA).

An equal amount of protein (30 μg) was loaded into each lane of a 10% SDS-polyacrylamide gel. (Based on an average lysate concentration of 6 μg/μL, 4.9 μL of lysate was mixed with loading buffer to a final 20 μL volume). Electrophoresis was conducted at 90 V for 120 min. Proteins were subsequently transferred to a nitrocellulose membrane.

The membranes were blocked for 1 h at room temperature in 5% non-fat skim milk (in TBS-T) and then washed three times (10 min each) with TBS-T. Membranes were incubated with primary antibodies for 1 h. Following another wash cycle, they were incubated with the appropriate HRP-conjugated secondary antibody (in 5% skim milk) for 2 h at room temperature. Protein bands were visualized using an enhanced chemiluminescence (ECL) substrate and captured on a ChemiDoc XRS + imaging system (Bio-Rad, Hercules, CA, USA). The primary and secondary antibodies used for Western blotting, including their dilution ratios, sources, and catalog numbers, are listed in Table S2.

Due to the highly limited volume of protein extracts available from the specific brain subregions, it was unfeasible to probe all target proteins and loading controls on a single membrane. To strictly ensure equal loading across different targets, identical protein master mixes from the same sample sets were simultaneously loaded in equal amounts onto parallel gels and run under identical electrophoretic conditions. Consequently, a single representative β-actin blot, run in parallel with the same master mix, was utilized as the standard loading control for normalization.

### Immunohistochemistry analyses

Paraffin blocks were sectioned at a thickness of 4 μm, mounted onto slides, and dried at room temperature, followed by incubation at 65 °C for 2 h. Prior to staining, the sections underwent deparaffinization and rehydration. Endogenous peroxidase activity was quenched by incubating the slides in hydrogen peroxide for 10 min at room temperature, followed by rinsing with PBS-T (phosphate-buffered saline with 0.1% Tween-20).

The sections were then incubated overnight at 4 °C with primary antibodies. Following this, slides were treated with an anti-rabbit/mouse HRP-conjugated secondary antibody (Agilent, #K5007, Santa Clara, CA, USA) for 30 min at room temperature. The chromogenic signal was developed using DAB (Agilent Dako, #K5007). The development time was 3 min for all markers. Finally, sections were rinsed with PBS-T, counterstained with Mayer's Hematoxylin (MUTO PURE CHEMICALS, #30002, Tokyo, Japan) for 4 min, and imaged using a light microscope.

To stain Aβ plaques, Thioflavin S was applied to the sections for 10 min at room temperature. Images were captured using a Nikon TS2-S-SM microscope equipped with a Nikon DS-Qi2 camera (Nikon Microscopy, Tokyo, Japan).

The primary and secondary antibodies used for immunohistochemistry, including their dilution ratios, sources, and catalog numbers, are listed in Table S3. While thicker sections are often used for general plaque visualization, 4 μm thin sections were utilized in this study to prevent signal overlap in adjacent cells.

### Statistical analysis

All experimental data are expressed as the mean ± standard error of the mean (SEM). Statistical comparisons between experimental groups were performed using a one-way analysis of variance (ANOVA). When the ANOVA indicated a significant overall effect, Tukey's honestly significant difference (HSD) post-hoc test was used for multiple comparisons between individual groups. For the Morris Water Maze learning curve over training days, data were analyzed using a repeated measures ANOVA followed by Tukey's post-hoc test.

A *p*-value less than 0.05 was considered statistically significant. Significance levels are denoted in the figures as follows: ∗p < 0.05, ∗∗p < 0.01, ∗∗∗p < 0.005, and ∗∗∗∗p < 0.001.

## Result

### Vibrotactile stimulation (VTS) ameliorates cognitive deficits in 5xFAD mice

To evaluate whether VTS could ameliorate the cognitive decline observed in 5xFAD mice according to our experimental schedule (Fig. 1A), we conducted three behavioral tests: the Morris Water Maze (MWM), the Novel Object Recognition (NOR) test, and the Y-maze.

**Fig. 2:**
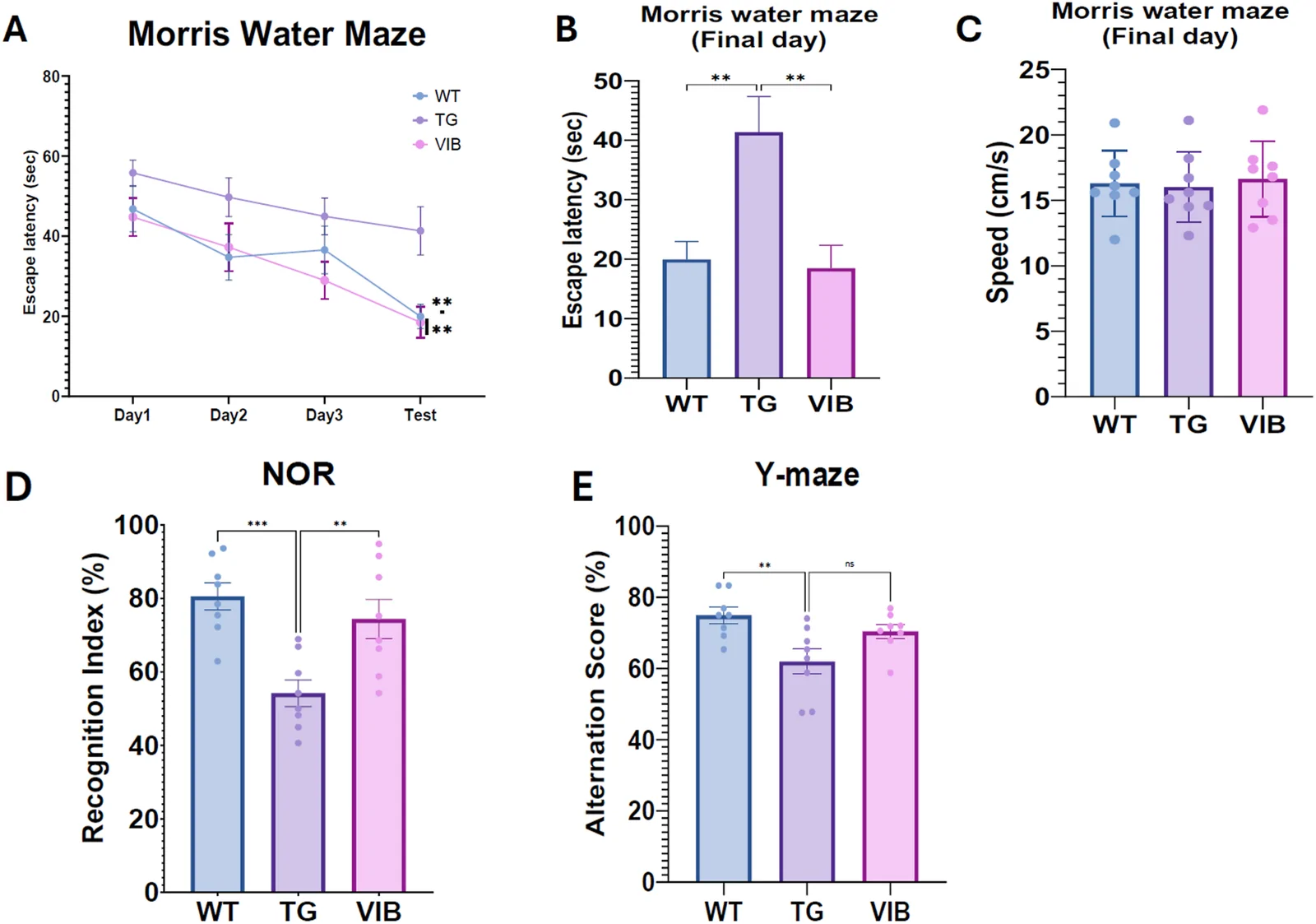
**Vibrotactile stimulation ameliorates cognitive deficits in 5xFAD mice.** (A) Mean daily escape latency during the 3-day training period of the Morris water maze (MWM) test. (B) Escape latency on the final day (Day 4). (C) Speed on the final day (Day 4). (D) Recognition Index (RI) in the novel object recognition (NOR) test, calculated as the ratio of time spent exploring the novel object to the total exploration time. (E) Percentage of spontaneous alternation in the Y-maze test. Data are presented as the mean ± SEM (n = 8 per group). ∗∗p < 0.01, ∗∗∗p < 0.005 vs. TG group. Statistical significance was determined by repeated measures ANOVA (for A) and one-way ANOVA (for B-E), followed by Tukey's post-hoc test.

In the MWM test, while all groups showed reduced escape latency over the training period, the TG group exhibited a significantly slower learning curve compared to the WT group (Fig. 2A), indicating severe spatial learning and memory impairment. When comparing the escape latency on the Test day (Day 4), both the WT and VIB groups showed significantly shorter latencies than the TG group (Fig. 2B). When comparing the speeds on the day of the test (Day 4), there appeared to be no statistically significant difference among the groups (Fig. 2C). This demonstrates that VTS significantly restored impaired spatial memory function in the 5xFAD mice.

In the NOR test, which assesses recognition memory, the VIB group displayed a significantly higher Recognition Index (RI) compared to the TG group. VTS treatment increased the RI by approximately 1.6-fold relative to the TG group, suggesting that VTS improved object recognition memory (Fig. 2D).

In the Y-maze test, the spontaneous alternation score did not differ significantly between the TG and VIB groups (Fig. 2E), indicating that VTS did not produce a detectable improvement in short-term spatial working memory under the present experimental conditions.

### VTS attenuates oxidative stress and cholinergic dysfunction in the cerebral cortex

**Fig. 3:**
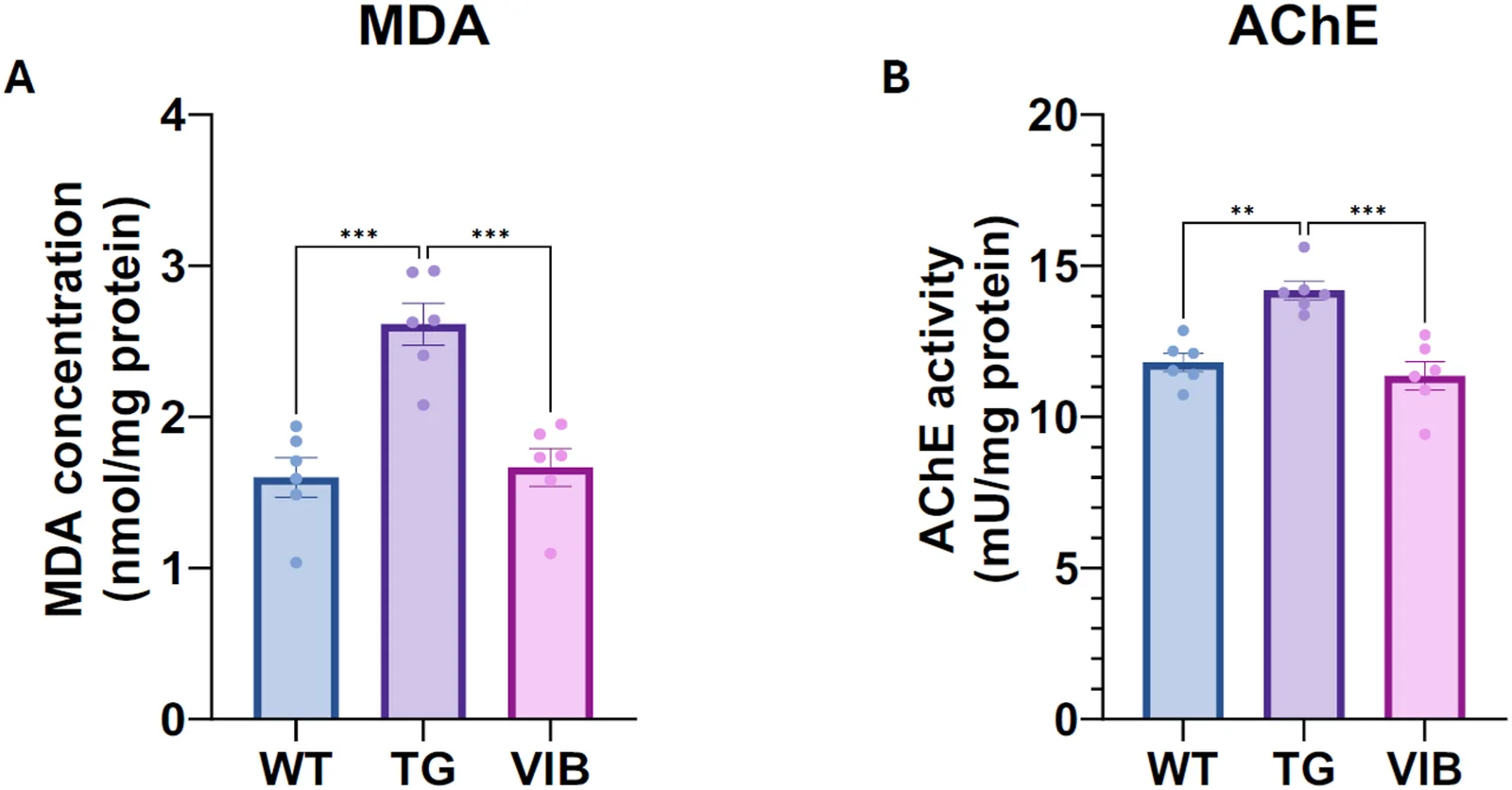
**Effects of vibrotactile stimulation on oxidative stress and AChE activity in 5xFAD mice.** (A) Cortical levels of malondialdehyde (MDA), a marker of lipid peroxidation and oxidative stress. (B) Acetylcholinesterase (AChE) activity in the cortex. Data are presented as the mean ± SEM (n = 6 per group). ∗p < 0.05, ∗∗p < 0.01, vs. TG group. Statistical significance was determined by one-way ANOVA followed by Tukey's post-hoc test.

To elucidate the biochemical mechanisms by which VTS improves cognition in 5xFAD mice, we analyzed markers of oxidative stress and cholinergic neurotransmission in the cerebral cortex. Oxidative stress was assessed by quantifying the levels of malondialdehyde (MDA), a primary end-product of lipid peroxidation. The TG group exhibited significantly elevated MDA levels compared to the WT group, confirming a state of heightened oxidative stress associated with AD pathology (Fig. 3A). Notably, VTS treatment significantly attenuated this increase. The VIB group showed a significant reduction in MDA levels compared with the TG group, suggesting that VTS effectively mitigates oxidative stress in the 5xFAD brain (Fig. 3A).

Cholinergic system integrity was evaluated by measuring acetylcholinesterase (AChE) activity. The TG group displayed significantly heightened AChE activity relative to the WT group, indicating cholinergic dysfunction (Fig. 3B). Conversely, VTS treatment counteracted this deficit. The VIB group showed a significant decrease in AChE activity compared to the TG group, restoring it to a level comparable to that of the WT controls (Fig. 3B).

### VTS suppresses amyloidogenesis and promotes Aβ clearance in the hippocampus

To determine whether VTS directly impacts Aβ accumulation in 5xFAD mice, we performed histochemical staining and Western Blot analysis on hippocampal tissue.

**Fig. 4:**
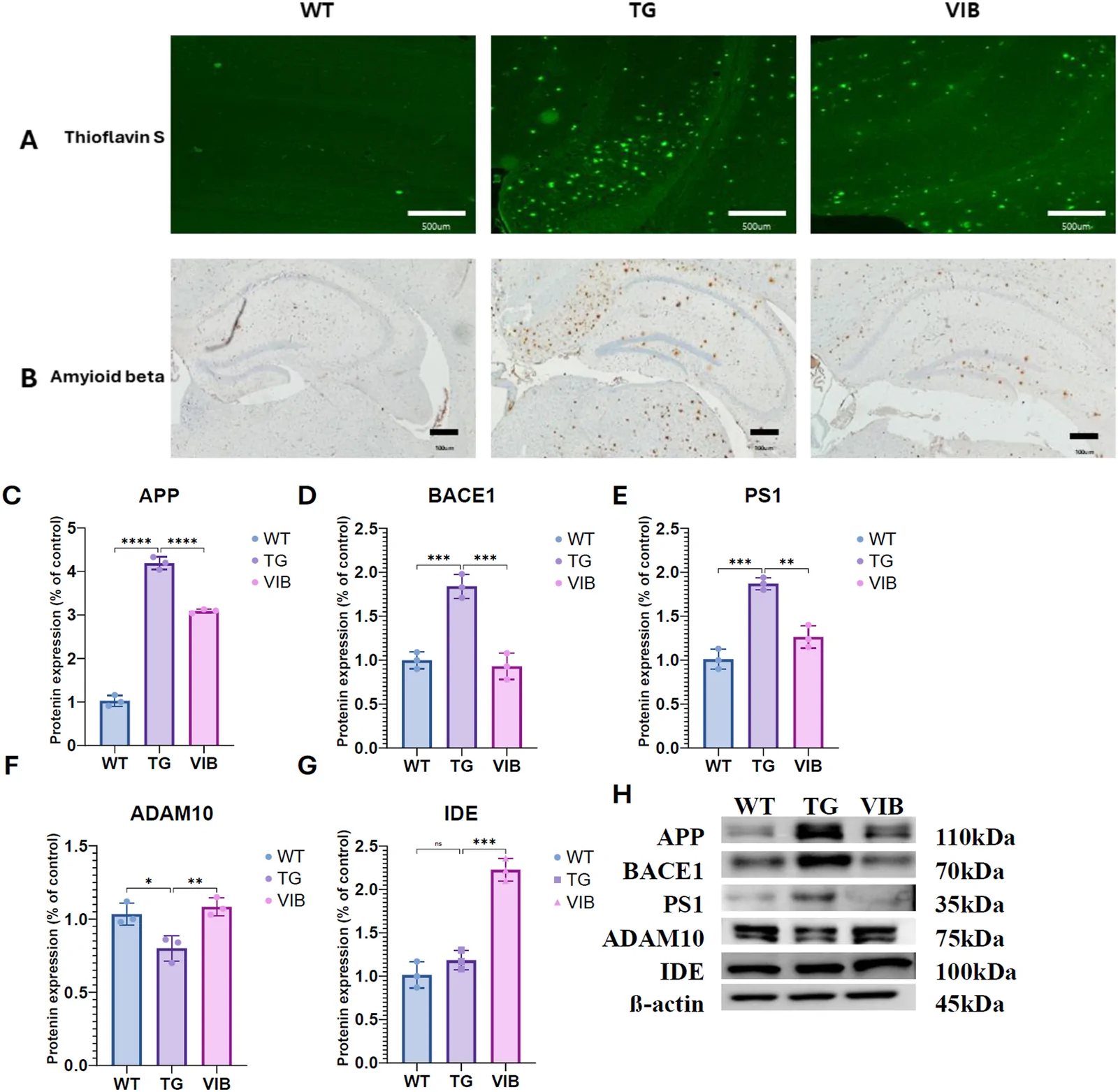
**Vibrotactile stimulation reduces amyloid-beta pathology and modulates APP processing pathways in the hippocampus of 5xFAD mice.** (A) Representative fluorescence images of Thioflavin S staining for dense-core plaques in the hippocampus (x100). (B) Representative images of Aβ immunohistochemistry staining in the hippocampus (x40). (C–G) Quantitative analysis of hippocampal protein levels for (C) APP, (D) BACE1, (E) PS1, (F) ADAM10, and (G) IDE. All protein levels were normalized to β-actin. (H) Representative Western blot bands showing the expression of APP, BACE1, PS1, ADAM10, IDE, and β-actin (loading control) for the quantification shown in (C-G) Scale bar (A) 500 μm, (B) 100 μm. Data are presented as the mean ± SEM (n = 3 per group). ∗p < 0.05, ∗∗p < 0.01, vs. TG group. Statistical significance was determined by one-way ANOVA followed by Tukey's post-hoc test.

First, we assessed the Aβ plaque burden histologically. Extensive Aβ deposition was observed in the hippocampal regions of the TG group. Thioflavin S staining, which visualizes dense-core plaques, revealed that the VTS-treated VIB group had a significant reduction in both plaque number and area compared to the TG group (Fig. 4A). Consistent with this finding, Aβ immunohistochemistry (IHC), which directly targets the Aβ peptide, also confirmed that the total Aβ deposition was markedly decreased in the VIB group relative to the TG group (Fig. 4B).

To investigate the mechanisms underlying this plaque reduction, we quantified the expression of key proteins involved in Aβ production and clearance via Western Blot. The expression of key amyloidogenic pathway components, including APP, BACE1, and PS1, was significantly upregulated in the TG group compared to the WT group. Notably, VTS treatment significantly reversed these increases, as the VIB group exhibited markedly reduced expression of all three proteins compared to the TG group (Fig. 4C–E).

Conversely, the expression of ADAM10, which initiates the non-amyloidogenic pathway, was decreased in the TG group but was significantly upregulated by VTS treatment (Fig. 4F). Furthermore, we observed that the expression of IDE, a primary Aβ-degrading enzyme, was also significantly elevated in the VIB group compared to the TG group (Fig. 4G).

ELISA analysis demonstrated that PBS-soluble Aβ40 and Aβ42 levels were significantly elevated in the TG group compared with the WT group. Notably, VTS significantly reduced both PBS-soluble Aβ40 and Aβ42 levels compared with the TG group (Fig. S1).

These biochemical findings, together with the reductions in Aβ immunoreactivity and Thioflavin S-positive plaque burden, indicate that VTS reduced multiple measures of Aβ pathology.

### VTS is associated with alterations in hippocampal Piezo1, ERK, GSK-3β, and p65 signaling

To elucidate the molecular changes associated with the effects of VTS, we analyzed the expression and phosphorylation state of proteins involved in key hippocampal signaling pathways (ERK, GSK3β/p65).

**Fig. 5:**
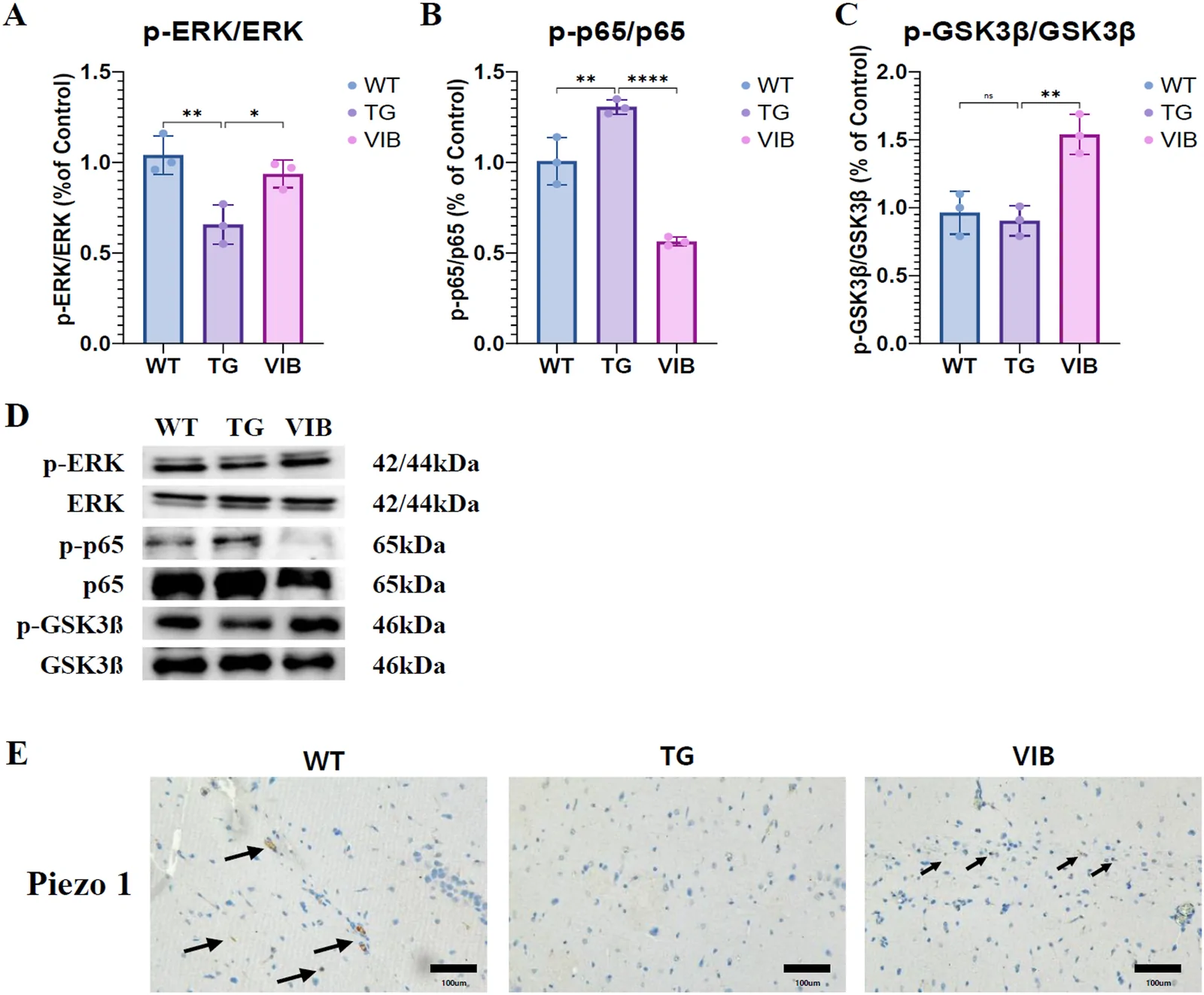
**Effects of vibrotactile stimulation on Piezo1/ERK and Piezo1/GSK-3β/p65 pathways in the hippocampus of a 5xFAD mouse model** (A-C) Quantitative analysis of hippocampal protein levels for (A) *p*-ERK/ERK, (B) p-p65/p65, and (C) *p*-GSK-3β/GSK-3β, (D) Representative Western blot bands showing the expression of *p*-ERK, ERK, *p*-GSK-3β, GSK-3β, p-p65, p65 for the quantification shown in (A–C). (E) Representative immunohistochemistry (IHC) staining images for Piezo1 in the hippocampus (x100). Scale bar = 100 μm. Data are presented as the mean ± SEM (n = 3 per group). ∗∗∗p < 0.005, ∗∗∗∗p < 0.001 vs. TG group. Statistical significance was determined by one-way ANOVA followed by Tukey's post-hoc test.

First, we observed that the expression of *p*-ERK, which was reduced in the TG group, was significantly restored in the VIB group following VTS treatment (Fig. 5A).

Next, we investigated the GSK3β/p65 pathway. VTS treatment led to a significant 1.5-fold increase in the inhibitory phosphorylation of GSK3β (*p*-GSK3β at Ser9) compared to the TG group. Consistent with this inhibition of GSK3β, the expression of p-p65, the activated form of the inflammatory transcription factor NF-κB, was significantly decreased by 2.0-fold in the VIB group relative to the TG group (Fig. 5 B, C).

To identify a potential mechanosensitive component associated with the response to VTS, we analyzed the expression of the mechanosensitive ion channel Piezo1 via immunohistochemistry. We found that Piezo1 expression, which was reduced in the TG group, was restored in the VIB group to levels comparable to WT controls (Fig. 5E).

The hippocampal p-tau/total tau ratio was significantly increased in the TG group compared with the WT group. This increase was significantly attenuated in the VIB group (Supplementary Fig. S2), indicating that VTS reduced tau phosphorylation in 5xFAD mice. Collectively, VTS treatment was accompanied by increased Piezo1 expression, increased *p*-ERK expression, increased inhibitory phosphorylation of GSK-3β at Ser9, and decreased p-p65 expression. These concurrent changes identify a candidate Piezo1-associated signaling framework.

### VTS attenuates neuroinflammation and neuronal loss in 5xFAD mice

**Fig. 6:**
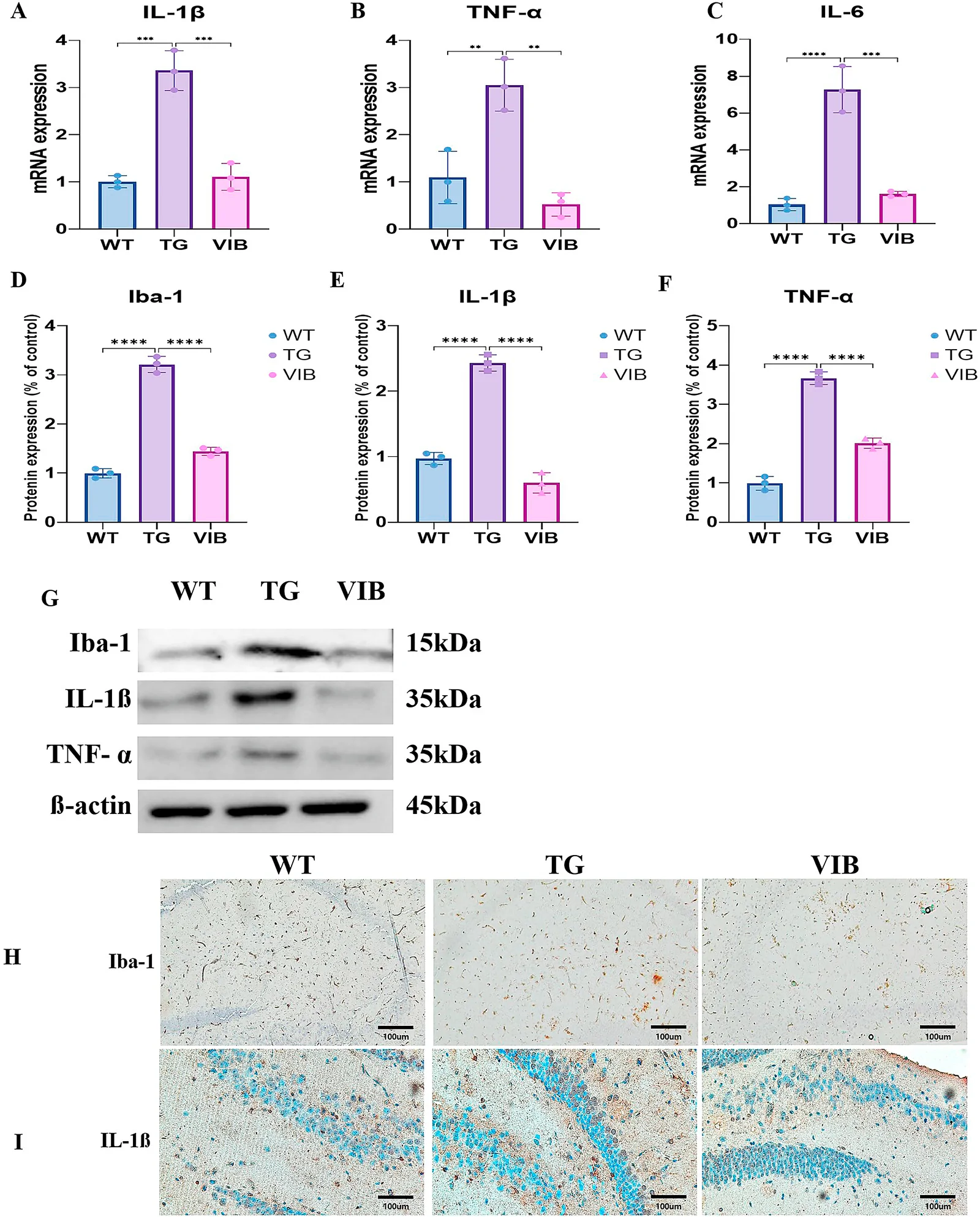
**Vibrotactile stimulation attenuates systemic inflammation and neuroinflammation in 5xFAD mice.** (A–C) Relative mRNA expression levels of pro-inflammatory cytokines (A) IL-1β, (B) TNF-α, and (C) IL-6 in the blood, determined by qRT-PCR analysis. (D–F) Quantitative analysis of hippocampal protein levels for (D) Iba-1, (E) IL-1β, and (F) TNF-α, normalized to β-actin. (G) Representative Western blot bands showing the expression of Iba-1, IL-1β, TNF-α, and β-actin (loading control) for the quantification shown in (D–F). (H, I) Representative immunohistochemistry (IHC) staining images for (H) Iba-1 (a microglial marker) and (I) IL-1β in the hippocampus (x100). Scale bar = 100 μm. Data are presented as the mean ± SEM (n = 3 per group). ∗∗∗p < 0.005, ∗∗∗∗p < 0.001 vs. TG group. Statistical significance was determined by one-way ANOVA followed by Tukey's post-hoc test.

First, we assessed the systemic inflammatory status. Analysis of inflammatory cytokines (IL-1β, TNF-α, IL-6) in the blood showed that their levels were significantly elevated in the TG group, and VTS treatment significantly reduced the levels of these cytokines in the VIB group (Fig. 6A–C).

Next, we investigated the neuroinflammatory status in the hippocampus using Western Blot quantification. Compared to the WT group, the TG group showed significant upregulation of the inflammatory markers Iba-1 (approx. 3.0-fold), IL-1β (approx. 2.5-fold), and TNF-α (approx. 3.5-fold). Notably, VTS treatment effectively counteracted this neuroinflammation; the VIB group displayed significant reductions in Iba-1 (approx. 2.8-fold), IL-1β (approx. 2.6-fold), and TNF-α (approx. 1.8-fold) expression compared to the TG group (Fig. 6D–F).

These quantitative findings were further corroborated by histological observations. IHC staining revealed that the TG group exhibited significant microglial activation (Iba-1) and elevated expression of the pro-inflammatory cytokine IL-1β. In contrast, the VTS-treated VIB group showed markedly reduced Iba-1 activation and diminished IL-1β expression (Fig. 6H and I).

**Fig. 7:**
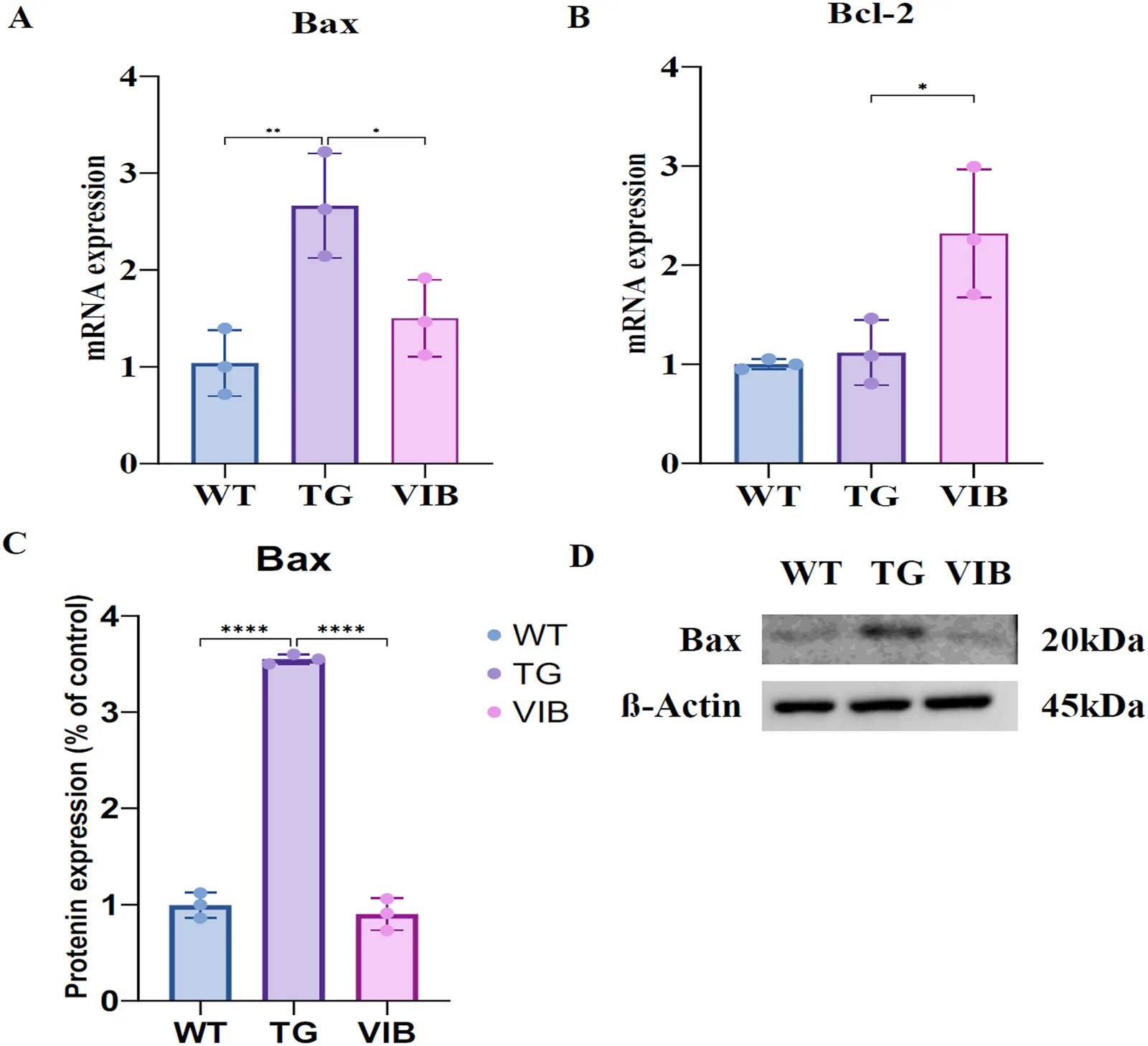
**Vibrotactile stimulation attenuates neuronal apoptosis in the hippocampus of 5xFAD mice.** (A, B) Relative mRNA expression levels of the apoptosis-related markers (A) Bax and (B) Bcl-2 in the hippocampus, determined by qRT-PCR analysis. (C) Quantitative analysis of Bax protein levels in the hippocampus, normalized to β-actin. (D) Representative Western blot bands showing the expression of Bax and β-actin (loading control) for the quantification shown in (C). Data are presented as the mean ± SEM (n = 3 per group). ∗p < 0.05, ∗∗p < 0.01, ∗∗∗p < 0.005 vs. TG group. Statistical significance was determined by one-way ANOVA followed by Tukey's post-hoc test.

Finally, we evaluated the impact of VTS on neuronal apoptosis. Gene expression analysis (qRT-PCR) in the hippocampus showed that the TG group had increased expression of the pro-apoptotic marker Bax (Fig. 7A). VTS treatment not only reduced the expression of Bax but also concurrently increased the expression of the anti-apoptotic marker Bcl-2 (Fig. 7B).

This shift at the genetic level was confirmed at the protein level. Western Blot analysis revealed that Bax protein expression was increased by approximately 3.5-fold in the TG group compared to the WT group, whereas VTS treatment suppressed Bax expression in the VIB group to a level comparable to that of WT controls (Fig. 7C).

### VTS modulates cholinergic and synaptic plasticity-associated markers in the hippocampus

To assess the impact of vibrotactile stimulation (VTS) on cholinergic dysfunction and synaptic loss, two key pathological features of AD, we utilized WB and IHC analyses in the hippocampus.

**Fig. 8:**
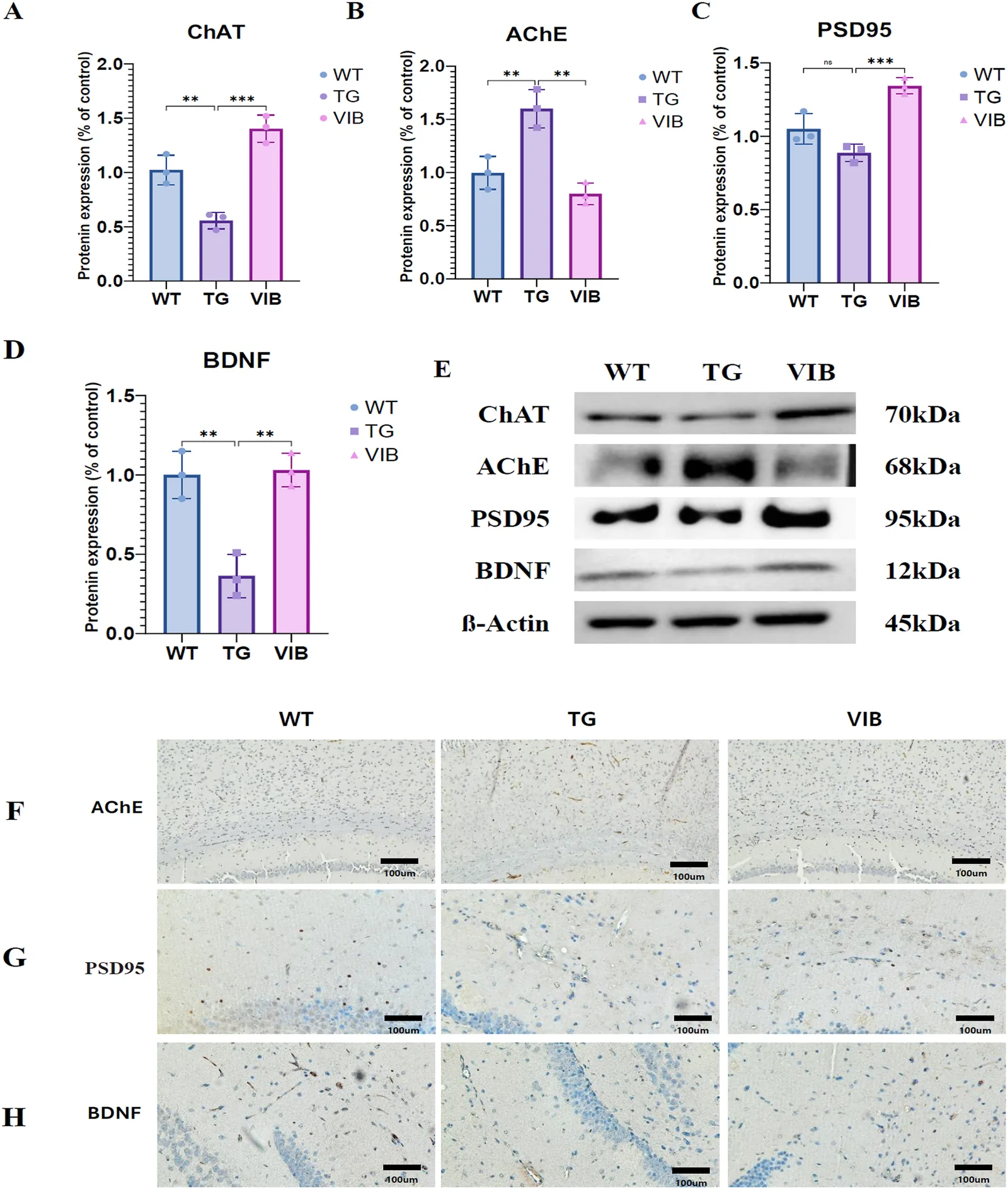
**Vibrotactile stimulation improves cholinergic neurotransmission and synaptic plasticity in 5xFAD mice**. (A–D) Quantitative analysis of hippocampal protein levels for (A) ChAT, (B) AChE, (C) PSD95, and (D) BDNF, normalized to β-actin. (E) Representative Western blot bands showing the expression of ChAT, AChE, PSD95, BDNF, and β-actin (loading control) for the quantification shown in (A–D). (F–H) Representative immunohistochemistry (IHC) staining images for (F) AChE, (G) PSD95 and (H) BDNF in the hippocampus (x100). Scale bar = 100 μm. Data are presented as the mean ± SEM (n = 3 per group). ∗∗∗p < 0.005, ∗∗∗∗p < 0.001 vs. TG group. Statistical significance was determined by one-way ANOVA followed by Tukey's post-hoc test.

First, we analyzed the expression of cholinergic and synaptic markers using quantitative Western Blot analysis. The expression of ChAT (Choline acetyltransferase), a marker for cholinergic neurons and the enzyme responsible for acetylcholine synthesis, was found to be significantly decreased in the TG group. VTS treatment successfully rescued this deficit, significantly increasing ChAT expression in the VIB group (Fig. 8A).

The WB results for AChE, BDNF, and PSD95 were also assessed. Specifically, the elevated AChE expression in the TG group was normalized in the VIB group, while the reduced levels of BDNF and PSD95 observed in the TG group were significantly increased following VTS treatment (Fig. 8B–D).

These quantitative findings were further validated by histological IHC analysis. IHC staining revealed that the TG group showed markedly upregulated AChE expression compared to the WT group, whereas VTS treatment significantly reduced AChE expression in the VIB group. Concurrently, the expression of BDNF and PSD95 were both significantly reduced in the TG group. Notably, VTS treatment restored the expression of both BDNF and PSD95 in the VIB group (Fig. 8F–H).

Taken together, these results indicate that VTS is associated with favorable changes in cholinergic and synaptic plasticity-associated protein expression, including ChAT, AChE, BDNF, and PSD95.

## Discussion

The present study evaluated the effects of 40 Hz vibrotactile stimulation on cognitive performance and AD-related neuropathological changes in male 5xFAD mice. VTS improved spatial learning performance and recognition memory, whereas no significant improvement was detected in Y-maze spontaneous alternation. These behavioral effects were accompanied by reduced hippocampal Aβ plaque burden and lower cortical soluble Aβ40 and Aβ42 levels, together with favorable changes in markers associated with amyloid processing and degradation, oxidative stress, neuroinflammation, cholinergic signaling, and synaptic integrity. VTS also increased hippocampal Piezo1 expression and altered ERK, GSK-3β, and p65 phosphorylation. Collectively, these findings support the potential of 40 Hz VTS as a non-invasive approach for ameliorating AD-related phenotypes and identify a candidate Piezo1-associated signaling framework that requires direct mechanistic validation.

Gamma waves (25–100 Hz), the fastest neural oscillations in the human brain, are known to play a critical role in cognitive functions, particularly within the hippocampus [[34], [35], [36]]. A significant reduction in this gamma activity has been observed in patients with AD, and this disruption has been shown to correlate with memory impairment [[37], [38], [39]].

According to recent studies, various sensory stimulation methods capable of restoring these gamma oscillations are being investigated [[40], [41], [42]]. It has been confirmed that non-invasive external stimuli, such as sensory stimulation, can successfully induce gamma entrainment. The 40 Hz stimulation frequency was selected based on previous studies of gamma-frequency sensory stimulation. Notably, 40 Hz whole-body vibration has been reported to elicit cortical gamma oscillations in rodents [31]. Consistent with the established behavioral phenotype of 5xFAD mice [33], untreated TG mice exhibited impairments in all behavioral tasks. VTS improved learning performance in the Morris water maze and cognitive memory in the novel object recognition test, but did not significantly improve spontaneous alternation in the Y-maze. These findings suggest that while the behavioral effects of VTS were clearly evident in spatial learning and cognitive memory, they did not apply to short-term spatial working memory under the investigated conditions. However, since EEG or electrophysiological recordings were not performed, it is impossible to determine whether the VTS protocol in this study induced brain gamma-wave synchronization. Therefore, these results should be interpreted as a behavioral response to 40 Hz VTS rather than as evidence of a mechanism dependent on gamma-wave synchronization.

The accumulation of Aβ plaques is a defining pathological hallmark in AD mouse models, and several studies have reported that sensory stimulation therapies can reduce this Aβ burden [24,30]. Similar to these reports, VTS reduced plaques in hippocampal Aβ immunostaining and Thioflavin S staining in 5xFAD mice. ELISA analysis showed that cortical Aβ40 and Aβ42 levels were also significantly lower in the VIB group than in the TG group, providing biochemical evidence for Aβ reduction.

To elucidate the mechanisms underlying this plaque reduction, we analyzed the expression of key proteins governing Aβ metabolism: APP, ADAM10, BACE1, PS1, and IDE.

VTS converted the APP processing pathway to a non-amyloid-producing pathway by showing increased ADAM10 expression and decreased levels of APP, BACE1, and PS1. In particular, the decrease in PS1 after VTS, despite PS1 being continuously overexpressed in 5xFAD mice, may reflect changes in post-translational stability associated with GSK-3β inhibition [43]. Similar modulation of amyloid-processing proteins has been reported in 5xFAD mice, where Aripiprazole increased ADAM10 while reducing APP and BACE1 [44], and *Trans*-Cinnamaldehyde decreased BACE1 and PS1 expression [45].

VTS promotes the non-amyloid process by increasing IDE expression, and also promotes Aβ degradation. In fact, IDE upregulation after hyperbaric oxygen therapy has been reported to be associated with a reduction in Aβ plaques in 5xFAD mice [46]. Taken together, these results suggest that VTS can reduce Aβ accumulation by promoting ADAM10-mediated non-amyloid production, inhibiting APP/BACE1/PS1-dependent amyloid production, and enhancing IDE-mediated Aβ clearance.

Piezo1 is a mechanosensitive ion channel that may connect physical stimulation to intracellular biochemical responses. Previous research using transcranial magneto-acoustic stimulation reported Piezo1-associated reductions in Aβ pathology and neuroinflammation in an AD mouse model, providing biological support for investigating Piezo1 in the response to mechanical stimulation [47].

We hypothesized that Piezo1 may be involved in the biochemical responses to VTS and therefore examined signaling molecules potentially associated with Piezo1. Our results from hippocampal immunostaining confirmed that Piezo1 expression was significantly upregulated in the VTS-treated group, a finding consistent with previous research demonstrating increased Piezo1 expression following mechanical stimulation.

GSK-3β and NF-κB are also implicated in amyloid-related signaling: GSK-3β can influence PS1 function and BACE1 expression [43,[48], [49], [50]], whereas NF-κB signaling regulates BACE1 transcription [51]. In addition, IDE contributes to extracellular Aβ degradation [52,53], and increased GSK-3 activity has been reported in association with AD-like molecular abnormalities resulting from impaired insulin signaling [54]. In the present study, VTS increased Piezo1 expression and the inhibitory phosphorylation of GSK-3β at Ser9 while decreasing p65 phosphorylation. These changes occurred concurrently with decreased BACE1 and PS1 expression and increased IDE expression. Experimental studies in other cellular systems provide support for separate associations between Piezo1 and these signaling molecules: PIEZO1 silencing attenuated fluid-shear-stress-induced AKT/GSK-3β signaling in osteoblastic cells [55], whereas pharmacological Piezo1 activation reduced p65 phosphorylation and NF-κB-dependent inflammatory responses in microglial cells [56]. The hippocampal p-tau/total tau ratio was also reduced following VTS, which is biologically consistent with the established involvement of GSK-3 signaling in tau phosphorylation [49,57]. Nevertheless, these concurrent changes do not establish a continuous Piezo1-GSK-3β-p65 pathway or demonstrate that the reduction in p-tau was mediated by GSK-3β. Rather, they identify a candidate Piezo1-associated signaling framework that requires direct pharmacological or genetic validation.

A second potential signaling response involves ERK and non-amyloidogenic APP processing. ERK/CREB signaling has been reported to promote ADAM10 expression and α-secretase-dependent APP processing [58,59], while mechanical stimulation or pharmacological Piezo1 activation can induce noncanonical EGFR signaling and subsequent ERK activation in other experimental systems [60]. In the present study, the VTS-associated increase in Piezo1 expression occurred concurrently with increased ERK phosphorylation and ADAM10 expression. These findings are consistent with the potential involvement of Piezo1-associated ERK signaling in the regulation of non-amyloidogenic APP processing. However, increased Piezo1 expression does not necessarily indicate channel activation, and neither Piezo1 nor ERK was directly inhibited or genetically manipulated. Therefore, the present data do not establish a sequential Piezo1-ERK-ADAM10 pathway but instead provide a biologically plausible framework for future mechanistic investigation.

Oxidative stress and chronic neuroinflammation are components of Alzheimer's disease pathology, and sustained microglia activation can increase neuronal damage [61,62]. Additionally, it has been reported that systemic inflammation is commonly elevated in patients with Alzheimer's disease [63,64]. In this study, VTS reduced MDA levels in the cortex, which was accompanied by a decrease in blood IL-1β, TNF-α, and IL-6 levels, as well as a decrease in the expression of Iba-1, IL-1β, and TNF-α in the hippocampus. These results are largely consistent with the reduction in inflammatory burden reported after PTH(1-34) treatment in 5xFAD mice and the reduction in inflammatory burden reported after whole-body vibration in stroke and traumatic brain injury models [[65], [66], [67], [68], [69]]. VTS reduced apoptosis markers by decreasing Bax expression and increasing Bcl-2 expression, which is similar to what has been reported after repetitive transcranial magnetic stimulation in experimental models of neurodegeneration and Aβ-related pathology [70,71]. Collectively, these results confirm that vibrotactile stimulation effectively reduces neuroinflammation and suppresses neuronal apoptosis.

BDNF contributes to neuronal survival and the maintenance of synaptic plasticity, and is associated with improved cognitive function in Alzheimer's disease models [72,73]. Cholinergic signaling is essential for learning and memory [74], and impaired cholinergic signaling is associated with cognitive impairment in 5xFAD mouse models [75,76]. Previous studies have shown that exposure to electromagnetic fields reduced AChE activity, while whole-body vibration increased hippocampal ChAT and Synaptophysin expression [77,78]. In this study, VTS reduced cortical AChE activity and hippocampal AChE expression. It also increased the expression of ChAT, BDNF, and PSD95 in the hippocampus. VTS exhibits beneficial effects on molecular markers associated with BDNF, synaptic integrity, and plasticity, providing a valid molecular context for the observed behavioral improvements. However, electrophysiological synaptic function was not measured. Therefore, the results of this study suggest that VTS helps promote BDNF expression and changes in indicators related to synaptic plasticity and cholinergic function.

This study has several limitations that should be considered when interpreting the findings. First, the absence of direct pharmacological or genetic manipulation of Piezo1 limits our ability to establish a causal relationship between Piezo1 and the effects of VTS in 5xFAD mice. Although the present findings and previous literature support separate associations between Piezo1 and ERK, GSK-3β, or NF-κB signaling, the current data remain correlative and do not establish a sequential Piezo1-ERK and Piezo1-GSK-3β-p65 signaling pathways in this model. Second, although eight animals per group were included in the behavioral experiments, the molecular and histological analyses were performed using smaller numbers of biological replicates (n = 2–3 per group) because the available brain tissue was allocated across Western blotting, qRT-PCR, and histological analyses. These limited sample sizes reduce statistical power and precision, while the use of parallel gels for Western blot analysis may have introduced inter-gel variability. Although the generally consistent direction of the changes across protein, transcript, and histological measurements provides convergent support for the observed molecular responses, these findings require validation in larger independent cohorts. Moreover, only male mice were examined; therefore, the findings cannot be generalized to female mice, and potential sex-dependent responses to VTS remain to be investigated. Finally, the biochemical assessment of Aβ was limited to soluble Aβ40 and Aβ42 in the cerebral cortex and did not quantify insoluble, fibrillar, or plaque-associated Aβ. Because the cortical ELISA and hippocampal histological analyses were conducted in different brain regions, the results cannot be directly extrapolated to total cerebral amyloid burden or used to distinguish among reduced Aβ production, altered aggregation, and enhanced clearance or degradation. Future studies incorporating direct manipulation of Piezo1, larger cohorts of both sexes, and region-matched quantification of soluble and insoluble Aβ species will be required to validate the proposed signaling framework and more comprehensively characterize the effects of VTS on AD-related pathology.

## Conclusion

The present study demonstrates that 40 Hz VTS improves spatial learning and recognition memory and reduces multiple measures of hippocampal Aβ pathology in male 5xFAD mice. These beneficial effects were accompanied by coordinated changes in oxidative stress, neuroinflammation, cholinergic function, and synaptic plasticity-associated markers. VTS also increased Piezo1 expression and altered ERK, GSK-3β, and p65 phosphorylation, together with changes in proteins involved in APP processing and Aβ degradation. Collectively, these findings support a candidate molecular framework in which Piezo1-associated signaling may contribute to the neuroprotective response to VTS, although the direct causal role of Piezo1 remains to be determined. Overall, this study expands our understanding of the biological responses induced by 40 Hz vibrotactile stimulation and supports its further development as a promising non-invasive strategy for ameliorating AD-related cognitive impairment and neuropathology.

## Ethics approval and consent to participate

All animal experimental procedures were approved by the Institutional Animal Care and Use Committee of Dongguk University (Approval number: IACUC- 2024-048-1). Not applicable for human subjects.

## Consent for publication

Not applicable.

## Availability of data and materials

All data generated or analyses during this study are included in this published article and its supplementary information files.

## Author contributions

• **Conceptualization:** Chang-Ho Shin, Jae-Young Ha, Myeong-Hyun Nam, and Young-Kwon Seo.
• **Methodology:** Chang-Ho Shin, and Myeong-Hyun Nam.
• **Data curation:** Myeong-Hyun Nam, Hee-Jung Park, and Zu-Yu Chen.
• **Formal analysis:** Myeong-Hyun Nam, Hee-Jung Park, Hee-Deok Yun, and Hee-Yeon Lee.
• **Investigation:** Myeong-Hyun Nam, Yuna Kim, Ju-Yeong Lee, and Hee-Yeon Lee.
• **Writing—original draft:** Myeong-Hyun Nam.
• **Writing—review and editing:** Young-Kwon Seo.
• **Supervision:** Young-Kwon Seo.
• **Project administration:** Young-Kwon Seo.
• **Funding acquisition:** Young-Kwon Seo.

All authors have read and agreed to the published version of the manuscript.

Investigation, Formal analysis, Validation, Data curation. Hee-Yeon Lee.

## Declaration of competing interests

C.-H.S. and J.-Y.H. are employees of AriBio Co., Ltd., a company developing vibrotactile stimulation devices. AriBio Co., Ltd. provided the vibrotactile stimulation device used in this study. The funders had no role in the collection, analysis, or interpretation of the data; in the writing of the manuscript; or in the decision to publish the results. The remaining authors declare no competing interests.
