# Supplementary material for: 40 Hz vibrotactile stimulation ameliorates amyloid pathology and cognitive deficits in 5xFAD mice and is associated with alterations in Piezo1, ERK, and GSK-3β/p65 signaling
Source: Neurotherapeutics. 2026 Sep 17;23(6):e01077. doi: 10.1016/j.neurot.2026.e01077 (PMC13602061; doi:10.1016/j.neurot.2026.e01077)
Supplement: Supplementary file 1 [file mmc1.pdf]

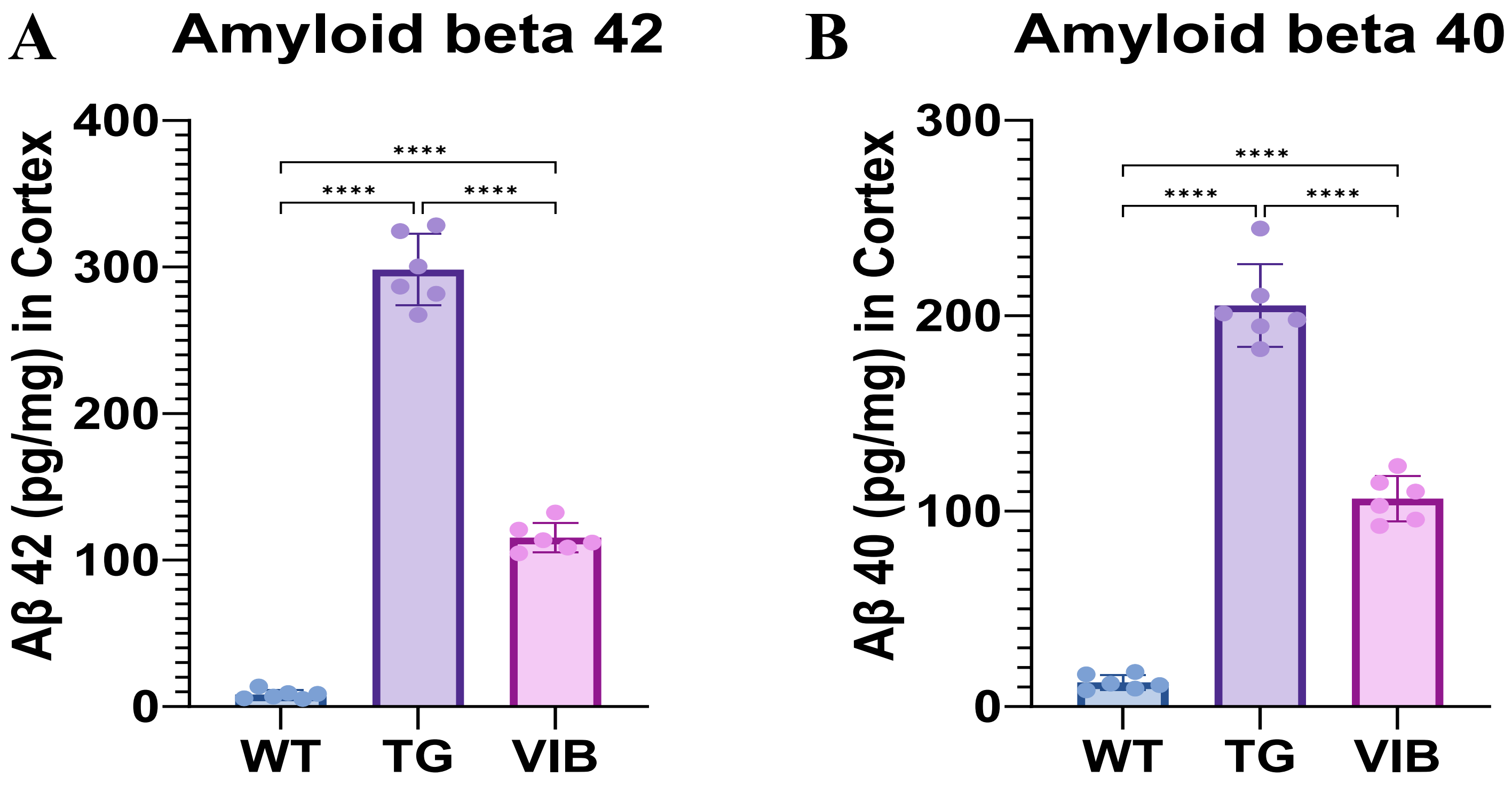

Supplementary Figure 1. Effects of 40 Hz vibrotactile stimulation on cortex amyloid beta 40, 42 in 5xFAD mice.

Amyloid-β (Aβ) peptide levels in the cerebral cortex. Cortical levels of (A) Aβ42 and (B) Aβ40 were quantified using ELISA. Data are presented as the mean ± SEM (n=6 per group). *** $p < 0.005$, **** $p < 0.001$ vs. TG group. Statistical significance was determined by one-way ANOVA followed by Tukey's post-hoc test.

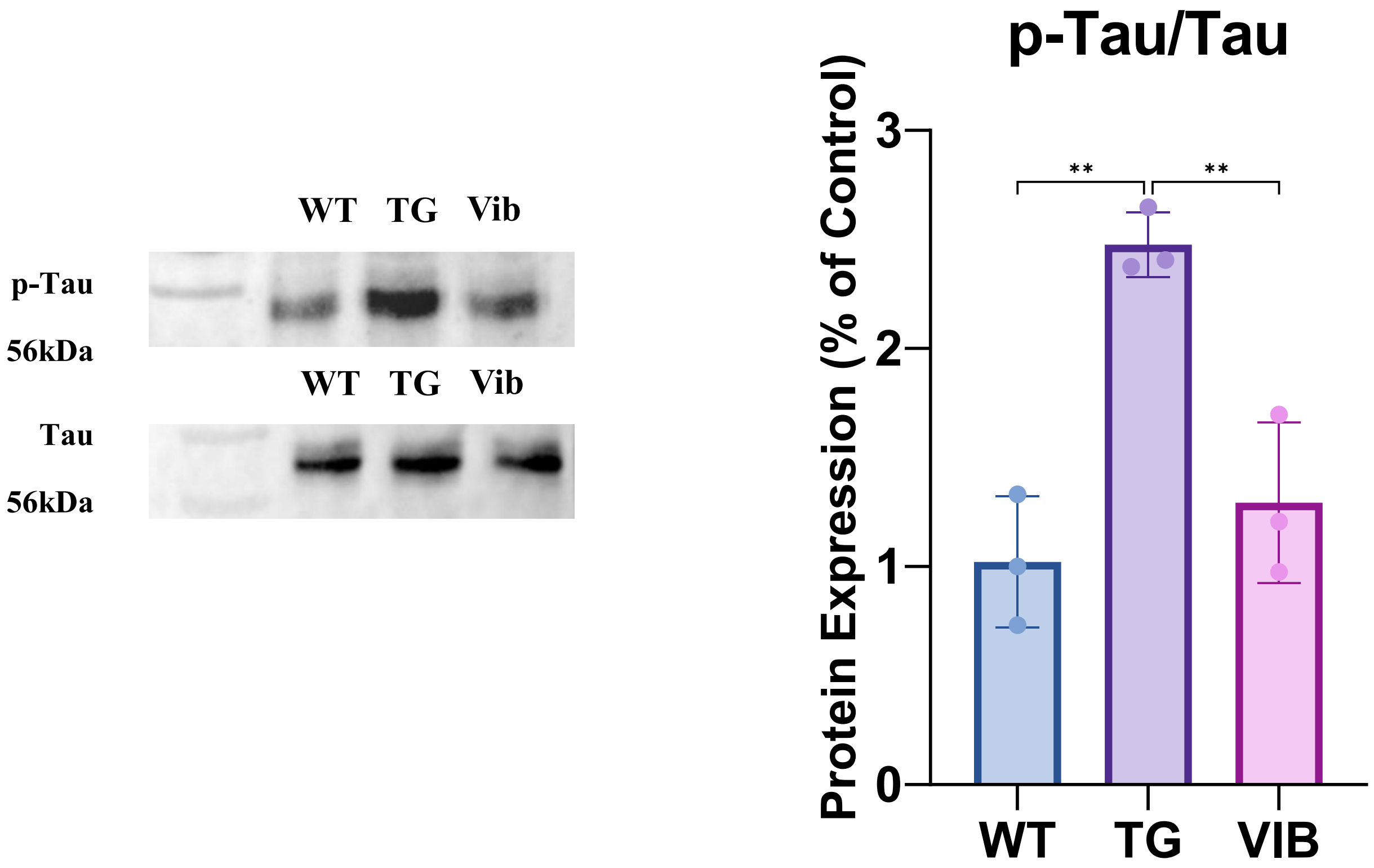

Supplementary Figure S2. Effects of 40 Hz vibrotactile stimulation on hippocampal tau phosphorylation in 5xFAD mice.

Representative Western blot images of p-tau (Ser199) and tau in the hippocampus. The graph presents the p-tau/ tau ratio. Data are presented as the mean ± SEM (n=3 per group). *** $p < 0.005$, **** $p < 0.001$ vs. TG group. Statistical significance was determined by one-way ANOVA followed by Tukey's post-hoc test.

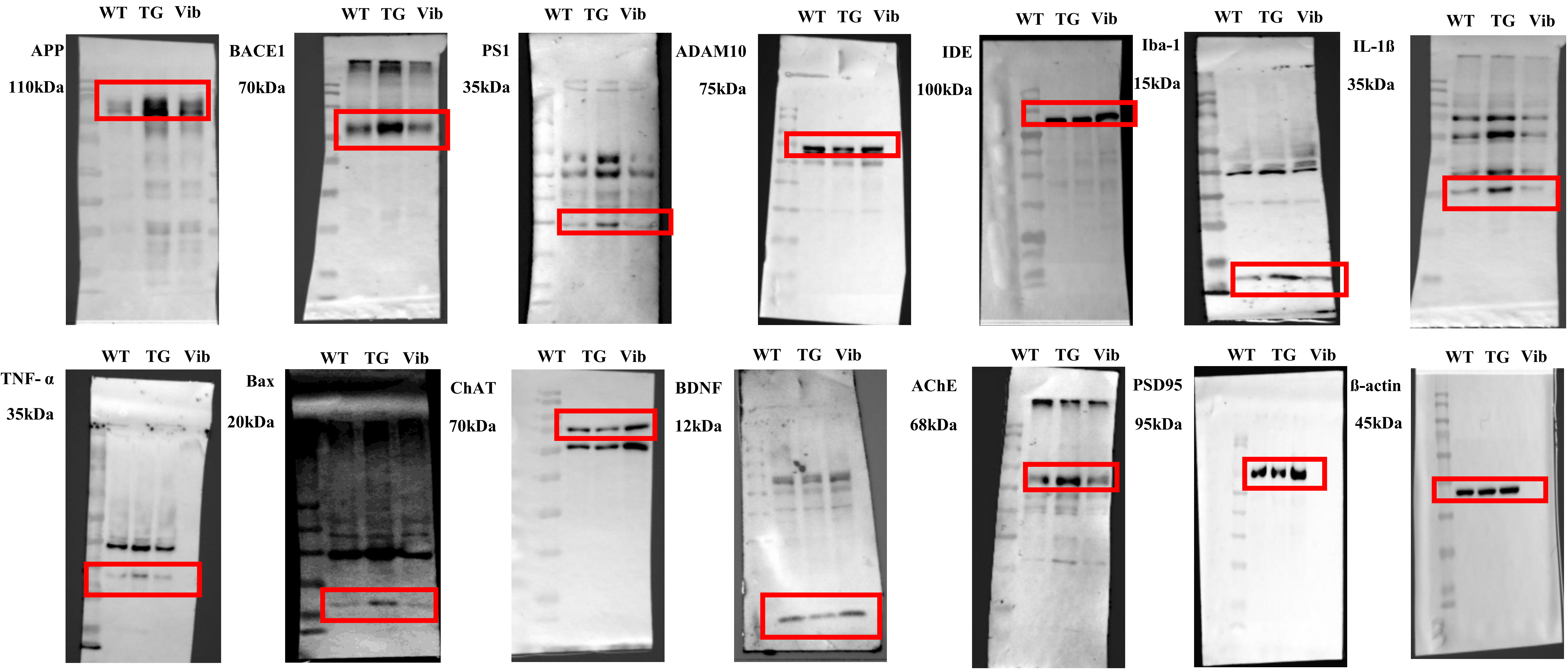

Supplementary Figure S3.

Original Uncropped Western blot image

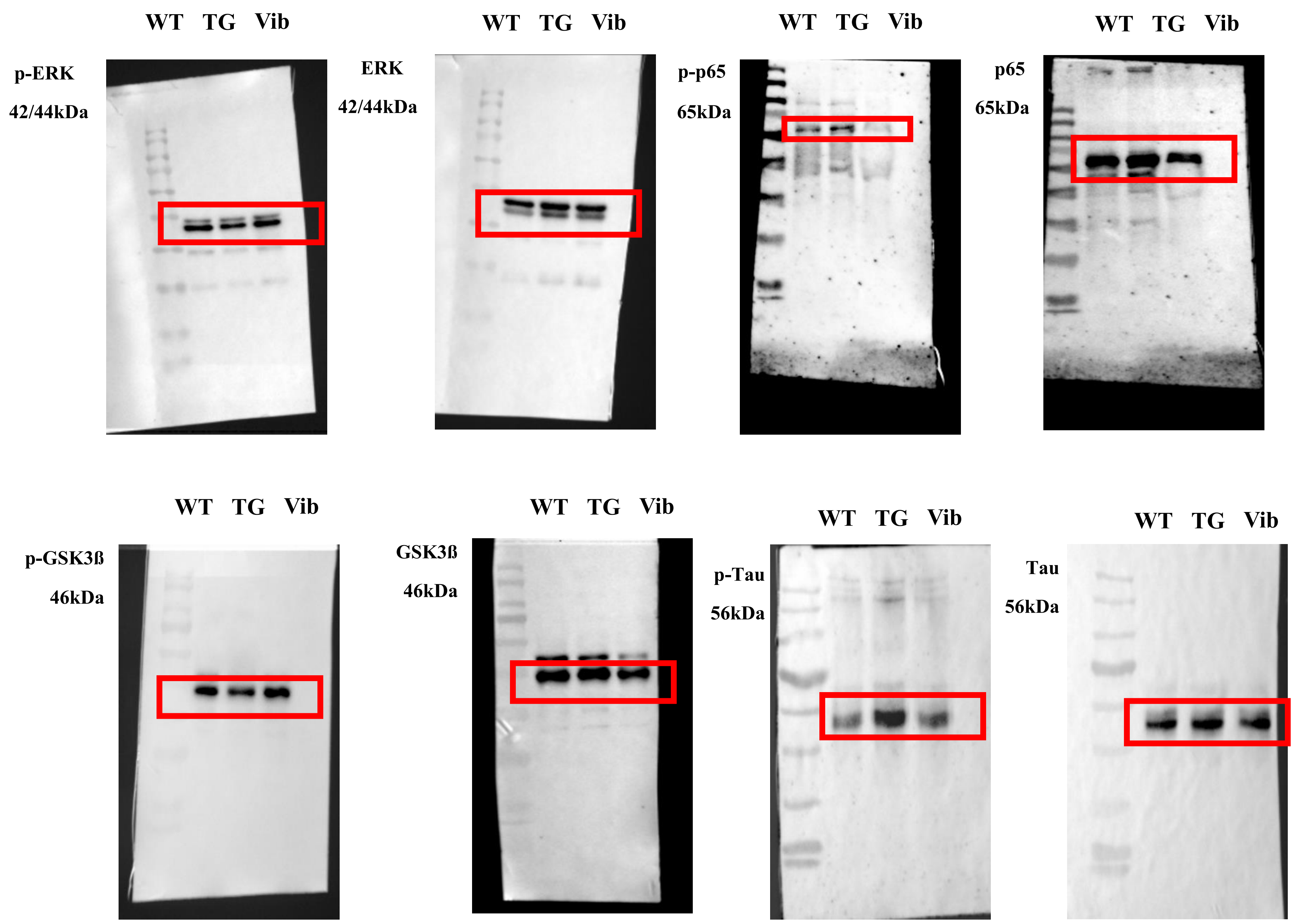

Supplementary Figure S3.
Original Uncropped Western blot image

Supplementary Table

Table S1. List of primer sequence

Gene	Forwad (5'-3')	Reverse(5'-3')
Mouse β-actin	AGGCCAACCGTGAAAAGATG	TGGCGTGAGGGAGAGCATAG
Mouse TNF-α	CACTCACAAACCACCAAGTG	GAGTAGACAAGGTACAACCC
Mouse IL-1β	TGCCACCTTTTGACAGTGATG	TGGGTGTGCCGTCTTTCATT
Mouse IL-6	CTG CAA GAG ACT TCCATC CAG TT	GAA GTA GGG AAG GCC GTG G
Mouse Bax	AAC ATC GCT CTG TGG ATG AC	GAG CAG CGT CTT CAG AGA CA
Mouse Bcl-2	GAATTGGCGATGAACTGGAC	GCAAAGTAGAAAAGGGCAACC

Table S2. List of primary and secondary antibodies used for Western blot analysis. Table includes antibody names, dilution ratios, sources (companies), and catalog numbers.

Antibody	Dilution	Company	Catalog Number
anti-β-actin	1:5,000	Abcam	ab8227
anti APP	1:500	Invitrogen	14-9749-82
anti BACE1	1:1,000	Cell Signaling	5606S
anti PS1	1:1,000	Abcam	ab76083
anti ADAM10	1:1,000	Abcam	ab1997
anti IDE	1:1,000	Abcam	ab32216
anti ERK	1:1,000	Cell Signaling	9102S
anti p-ERK	1:1,000	Cell Signaling	9101S
anti p65	1:1,000	Cell Signaling	8242S
anti p-p65	1:1,000	Cell Signaling	3033S
anti GSK3β	1:1,000	Cell Signaling	#9322
anti p-GSK3β	1:1,000	Cell Signaling	#9332
anti Iba-1	1:1,000	GeneTex	GTX635363
anti IL-1β	1:1,000	GeneTex	GTX74034
anti TNF-α	1:1,000	GeneTex	GTX110520
anti BAX	1:1,000	GeneTex	GTX109683
anti ChAT	1:1,000	Abclonal	A19031
anti AChE	1:1,000	GeneTex	GTX636298
anti PSD95	1:1,000	GeneTex	GTX133091
anti BDNF	1:1,000	GeneTex	GTX132621
anti Tau	1:1,000	Invitrogen	AHB0042
anti p-Tau	1:1,000	Invitrogen	701054
Goat anti-mouse IgG HRP	1:3,000	Abcam	ab6728
Goat anti-rabbit IgG HRP	1:3,000	Abcam	ab6721

Table S3. List of primary and secondary antibodies used for immunohistochemistry analysis. Table includes antibody names, dilution ratios, sources (companies), and catalog numbers.

Antibody	Dilution	Company	Catalog Number
anti β-amyloid	1:400	Invitrogen	44-344
anti Piezo 1	1:200	Novus Biologicals	NBP2-75617
anti Iba-1	1:400	GeneTex	GTX635363
anti IL-1β	1:200	GeneTex	GTX74034
anti AChE	1:200	GeneTex	GTX636298
anti PSD95	1:1,000	GeneTex	GTX133091
anti BDNF	1:200	GeneTex	GTX132621
anti-rabbit/mouse HRP	-	Agilent	K5007
